# Cross-cultural differences in men on active surveillance’ anxiety: a longitudinal comparison between Italian and Dutch patients from the Prostate cancer Research International Active Surveillance study

**DOI:** 10.1186/s12894-022-01062-z

**Published:** 2022-07-18

**Authors:** Paola Dordoni, Sebastiaan Remmers, Riccardo Valdagni, Lara Bellardita, Letizia De Luca, Fabio Badenchini, Cristina Marenghi, Monique J. Roobol, Lionne D. F. Venderbos

**Affiliations:** 1 Prostate Cancer Program, Fondazione IRCCS Istituto Nazionale dei Tumori, Milan, Italy; 2 Department of Urology, Erasmus Cancer Institute, Erasmus University Medical Center, Wytemaweg 80, kamer Na-1520, 3015 CN Rotterdam, The Netherlands; 3 Radiation Oncology, Fondazione IRCCS Istituto Nazionale dei Tumori, Milan, Italy; 4 Department of Oncology and Hemato-oncology, University of Milan, Milan, Italy

**Keywords:** Active surveillance, Prostate cancer, Anxiety, Cross cultural

## Abstract

**Background:**

Men diagnosed with localized prostate cancer (PCa) on active surveillance (AS) have shown to cope with anxiety caused by living with an ‘*untreated cancer*’ and different factors can influence the tolerance level for anxiety in these patients. The present study analyzes Italian (Milan) and Dutch (Rotterdam) men prospectively included in the Prostate cancer International Active Surveillance (PRIAS) trial, aiming to explore whether socio-demographic factors (i.e. age, relationship status, education, nationality) may be relevant factors in conditioning the level of anxiety at AS entry and over time.

**Methods:**

Italian and Dutch men participating in the IRB-approved PRIAS study, after signing an informed consent, filled in the Memorial Anxiety Scale for PCa (MAX-PC) at multiple time points after diagnosis. A linear mixed model was used to assess the relationship between the level of patient’s anxiety and time spent on AS, country of origin, the interaction between country and time on AS, patients’ relationship status and education, on PCa anxiety during AS.

**Results:**

823 MAX-PC questionnaires were available for Italian and 307 for Dutch men, respectively. Median age at diagnosis was 64 years (IQR 60–70 years) and did not differ between countries. On average, Dutch men had a higher total MAX-PC score than Italian men. However, the level of their anxiety decreased over time. Dutch men on average had a higher score on the PCa anxiety sub-domain, which did not decrease over time. Minimal differences were observed in the sub-domains PSA anxiety and fear of recurrence.

**Conclusion:**

Significant differences in PCa anxiety between the Italian and Dutch cohorts were observed, the latter group of men showing higher overall levels of anxiety. These differences were not related to the socio-demographic factors we studied. Although both PRIAS-centers are dedicated AS-centers, differences in PCa-care organization (e.g. having a multidisciplinary team) may have contributed to the observed different level of anxiety at the start and during AS.

*Trial registration* This study is registered in the Dutch Trial Registry (www.trialregister.nl) under NL1622 (registration date 11-03-2009), ‘PRIAS: Prostate cancer Research International: Active Surveillance—guideline and study for the expectant management of localized prostate cancer with curative intent’.

## Background

According to the international guidelines, men diagnosed with low-risk prostate cancer (PCa) can opt for definitive treatment (such as radical prostatectomy (RP) or radiotherapy (RT)), or opt for active surveillance (AS). AS involves a monitoring strategy with regular follow-up testing through prostate-specific antigen (PSA) tests, digital rectal examination (DRE), repeat prostate biopsies, and, when indicated, the use of magnetic resonance imaging (MRI). Should the PCa be reclassified or progress, then switching to RP or RT is still possible. Because the treatment choice for localized PCa is a preference-sensitive one, discussing quality of life (QoL) as part of shared decision making between the clinician and the patient is of central importance [1]. During this decision making process patients are supported by physicians and their family, and the decision making process involving all the stakeholders represents a valid tool for dealing with uncertainty and “emotional waves” [1, 2]. By choosing AS men may be aware that this is the choice that will likely protect their QoL, as it is the strategy that is related to the least side effects, mainly urinary incontinence, sexual dysfunction, and bowel inflammation [2–4].

Men on AS have 10- and 15-year disease-specific survival rates of > 98% and > 94% in long-running AS cohorts [5, 6]. While the disease-specific survival rates are excellent and studies show that AS has the least impact on prostate-specific functioning [7, 8], patients may be harmed by the psychological burden of living with an untreated cancer. Such burden has been found to be low for the majority of men following an AS-strategy. A small proportion of men on AS reported significant levels of anxiety after diagnosis but their anxious feelings decreased over time [9–13]. Even though the majority of studies in the literature agree that AS has limited impact on mental health and QoL [14], some studies reported different results [15–17]. Because the research evidence regarding the psychological impact of living with AS is mixed in its conclusions, in this study the focus will not lie on identifying whether or not men experience anxiety while on an AS management strategy [18]. Instead the aim is to explore whether socio-demographic factors (i.e. age, relationship status, education, and nationality) may be relevant factors in conditioning the level of anxiety at AS entry and over time in two dedicated AS-centers that are part of the Prostate cancer Research International Active Surveillance (PRIAS) study.

## Methods

### Study population

Men newly diagnosed with low-risk PCa, eligible for AS according to the PRIAS inclusion criteria—i.e. PSA ≤ 10 ng/mL; PSA-density < 0.2 ng/mL/mL; ≤ T2; 1–2 positive prostate needle-biopsy cores with Gleason score ≤ 3 + 3 = 6 between 2007–2017 and who signed a PRIAS-informed consent were invited to participate in the prospective QoL study. Men were eligible to participate in the QoL study if they spoke Italian or Dutch and were able to complete self-reported questionnaires. In the Italian center—since the introduction of the Memorial Anxiety scale for PCa (MAX-PC)—498 men have been enrolled in PRIAS. A total of 466 men were invited to participate in the QoL study; 32 men were not invited due to logistical reasons or an inability to complete the questionnaires. In the Netherlands the first 150 Dutch patients included in the PRIAS study were invited to participate in the QoL study. PRIAS (www.prias-project.org) and its associated prospective QoL study were approved by the Institutional Review Board of the Erasmus University Medical Center (PRIAS: MEC2004-339, PRIAS QoL: MEC2014-596) and of the Fondazione IRCCS Istituto dei Tumori in Milan (INT 46-07). Furthermore, all methods were carried out in accordance with relevant guidelines and regulations.

### Patient reported outcomes (PRO) measures

Men were invited to complete QoL-questionnaires at various timepoints; for the Italian center timepoints are at enrollment on AS (T0), 10 months after diagnostic biopsy (T1), immediately after the first re-biopsy (only patients still suitable for continuing on AS; T2), and then once a year after the first re-biopsy (T3); for the Dutch center timepoints are 3–6 months after enrollment on AS, 9 and 18 months after start on AS. The QoL-questionnaires contained validated measures to evaluate, amongst others, PCa-related anxiety (Memorial Anxiety Scale for PCa—MAX-PC) [19–22]. The MAX-PC includes 18 items, with for each item four response options (single item score range 0–3). The total MAX-PC score ranges from 0–54, with 54 indicating maximum PCa-related anxiety. Patients with scores of ≥ 27 were considered to have clinically significant PCa anxiety [19, 20]. The MAX-PC consists of three subscales that measure (1) general anxiety related to PCa and treatment—i.e. PCa anxiety, (2) anxiety related to PSA levels—i.e. PSA anxiety, and (3) fear of recurrence (fear of disease progression). Validated Italian and Dutch translations of the MAX-PC were used [21, 22].

### Statistical analyses

Linear mixed models were used to adjust for clustering at participant level using a random intercept for patients. Fixed effects included country (Italy or the Netherlands), education level (primary or secondary school; professional school or college; university and post-degree), relationship status (not married; married/living together), years on AS, and the interaction and its main effects between years on AS and country. The overall effect of a categorical variable was assessed by comparing the full imputed model with the model without the covariate of interest based on the likelihood ratio test.

All statistical analyses were performed with R version 3.5.1. Missing data were imputed using the Bayesian framework with R-package jointAI [23].

## Results

A total of 525 (395/466 Italian (response rate 84.8%%), 130/150 Dutch (response rate 86,7%)) men—who completed at least one QoL-questionnaire—were included in the analyses (Table 1). Median age at diagnosis was 64 years (IQR 60–70 years) and did not differ between countries. The maximum number of MAX-PC measurements was 823 for Italian and 307 for Dutch men.

**Table 1:** Patient characteristics

	Total cohort N = 525	PRIAS Milan, Italy N = 395	PRIAS Rotterdam, NL N = 130
Age, years (median, IQR)	64 (60–69)	65 (60–69)	64 (59–70)
Unknown (n)	19 (4%)	2 (0.5%)	17 (13%)
Education, n (%)			
Primary or secondary school	158 (30%)	87 (22%)	71 (55%)
Professional school or college	224 (43%)	183 (46%)	41 (32%)
University or post-degree	119 (23%)	103 (26%)	16 (12%)
Unknown	24 (5%)	22 (6%)	2 (2%)
Relationship status, n (%)			
Married/living together	447 (85%)	328 (83%)	119 (92%)
Not married	55 (10%)	45 (11%)	10 (8%)
Unknown	23 (4%)	22 (6%)	1 (1%)

### MAX-PC total score

The results of the analysis are presented in Table 2. The analysis shows that on average Dutch men score 13 points higher on the MAX-PC total score than Italian men (a higher score refers to a higher level of anxiety). In addition, the score decreases for Dutch men with on average one point for every year on AS compared to change over time for Italian men. These differences were not related to relationship status and education.

**Table 2:** Results of the model predicting the MAX-PC total score

Variable	Coefficient (95% CI)	*p* value
Intercept	0.92 (− 0.38 to 2.23)	0.16
Country		
Italy	Reference	
The Netherlands	13.11 (12.09–14.13)	< 0.001
Education		0.5
Primary or Secondary school	Reference	
Professional school or College	− 0.53 (− 1.41 to 0.34)	
University and post-degree	− 0.42 (− 1.44 to 0.59)	
Relationship status		0.6
Not married	Reference	
Married/living together	0.3 (− 0.86 to 1.46)	
Years on active surveillance (years)	0.2 (− 0.36 to 0.76)	0.5
Years on active surveillance (years) for Italian men	Reference	
Years on active surveillance (years) for Dutch men	− 0.95 (− 1.72 to − 0.17)	0.017

### MAX-PC subscale ‘PCa anxiety’

The results of the analysis are presented in Table 3 and show that on average Dutch men score nine points higher on the MAX-PC subscale ‘PCa anxiety’ than Italian men. The score for Dutch men does not decrease over time compared to the change over time for Italian men. Relationship status and education are no predictors for the differences seen in the ‘PCa-anxiety’ subscale scores.

**Table 3:** Results of the model predicting the MAX-PC ‘PCa anxiety’ subscale score

Variable	Coefficient (95% CI)	*p* value
Intercept	0.78 (− 0.25 to 1.81)	0.14
Country		
Italy	Reference	
The Netherlands	8.63 (7.83–9.43)	< 0.001
Education		0.8
Primary or Secondary school	Reference	
Professional school or College	− 0.25 (− 0.93 to 0.44)	
University and post-degree	− 0.25 (− 1.05 to 0.55)	
Relationship status		0.5
Not married	Reference	
Married/living together	0.28 (− 0.63 to 1.19)	
Years on active surveillance (years)	− 0.08 (− 0.51 to 0.35)	0.7
Years on active surveillance (years) for Italian men	Reference	
Years on active surveillance (years) for Dutch men	− 0.11 (− 0.71 to 0.48)	0.7

### MAX-PC subscale ‘PSA Anxiety’

On average, Dutch men score 0.5 points lower on the MAX-PC subscale ‘PSA anxiety’ than Italian men. This subscale score for Dutch men does not decrease over time as compared to the change over time for Italian men, see Table 4. Relationship status and education are no predictors for the differences seen in the ‘PSA anxiety’ subscale scores.

**Table 4:** Results of the model predicting the MAX-PC ‘PSA anxiety’ subscale score

Variable	Coefficient (95% CI)	*p* value
Intercept	0.85 (0.60–1.09)	< 0.001
Country		
Italy	Reference	
The Netherlands	− 0.53 (− 0.73 to − 0.33)	< 0.001
Education		0.2
Primary or Secondary school	Reference	
Professional school or College	0.08 (− 0.08 to 0.24)	
University and post-degree	0.17 (− 0.02 to 0.36)	
Relationship status		0.4
Not married	Reference	
Married/living together	− 0.09 (− 0.30 to 0.13)	
Years on active surveillance (years)	0.11 (− 0.01 to 0.23)	0.080
Years on active surveillance (years) for Italian men	Reference	
Years on active surveillance (years) for Dutch men	− 0.03 (− 0.21 to 0.14)	0.7

### MAX-PC subscale ‘Fear of Recurrence’

On average, Dutch men score three points higher on the MAX-PC subscale ‘Fear of Recurrence’ than Italian men. This subscale score decreases for Dutch men, with on average 0.7 points for every additional year on AS compared to the change over time for Italian men, see Table 5. Relationship status and education are no predictors for the differences seen in the ‘Fear of recurrence’ subscale scores.

**Table 5:** Results of the model predicting the MAX-PC ‘Fear of recurrence’ subscale score

Variable	Coefficient (95% CI)	*p* value
Intercept	1.06 (0.65–1.48)	< 0.001
Country		
Italy	Reference	
The Netherlands	3.38 (3.01–3.74)	< 0.001
Education		0.3
Primary or Secondary school	Reference	
Professional school or College	− 0.20 (− 0.46 to 0.06)	
University and post-degree	− 0.15 (− 0.45 to 0.15)	
Relationship status		0.4
Not married	Reference	
Married/living together	− 0.11 (− 0.46 to 0.24)	
Years on active surveillance (years)	0.03 (− 0.21 to 0.27)	0.8
Years on active surveillance (years) for Italian men	Reference	
Years on active surveillance (years) for Dutch men	− 0.66 (− 1.03 to − 0.30)	< 0.001

## Discussion

The present study aims to identify cultural-related factors associated with psychological well-being addressing anxiety as the main outcome in patients undergoing AS as a treatment for localized PCa in two dedicated AS-centers that are part of PRIAS.

Findings showed significant differences between the Italian and Dutch cohorts in self-reported disease-specific anxiety, the latter group of men showing a higher overall level of disease-specific anxiety of 13 points as measured by the MAX-PC questionnaire. Results of the analyses showed that the significant differences in disease-specific anxiety in our study could not be explained by sociodemographic factors such as education or relationship status, nor by the relation between center and years on AS, and the time on AS (in years). Country seems to be the variable that is associated with the observed levels of anxiety.

In a study by Ruane-McAteer and colleagues, AS patients showed higher anxiety levels compared to patients undergoing active treatment within nine months after diagnosis. Authors suggested that anxiety levels might be lower among patients undergoing AS at medical centres with a long experience in managing patients on AS. It is possible that focusing on studying and refining their AS protocols and procedures may lead to a greater acceptance and trust in AS by both patients and their clinicians [17]. That does not necessarily hold that collaborating specialists in the field of urology, like the oncologists and radiotherapists from the same centre, share such an attitude. In this study, the QoL-data from two dedicated AS-centers which follow the same AS-protocol have been analyzed. While we can expect differences in the psychological well-being of patients on AS between AS-dedicated vs. non-dedicated centers, here it is seen that even between two AS-dedicated centers the level of disease-specific anxiety can differ significantly. It can be hypothesized that cultural differences in cancer coping strategies between Northern and Southern European populations may play a role, but it might also be that this finding can be explained, in part, by the different clinical settings adopted in the two organizations. In the qualitative research conducted by Seaman et al., the importance of the clinician–patient relationship in navigating the experience of a PCa diagnosis and subsequent management with AS is shown [17, 24]. Before entering on AS, Italian patients meet with the different specialists involved in PCa care within a multidisciplinary team *simultaneously* (i.e. a urologist, a radiation oncologist and a psychologist), and they discuss the available treatment options together in one session (i.e. RP, external beam radiotherapy (EBRT), brachytherapy (BT) and AS). The multidisciplinary team shows men a unique agreement about “*what he can do*” and navigates with the patients and his family through the different options so that the patient is proactive in the final decision of the ‘best’ strategy for himself, in that particular moment of his life [1]. In this context, men may feel reassured that whatever treatment choice they make is supported by the multidisciplinary team of specialists and hence experience a feeling of hope about the possibility of protecting their QoL. Furthermore, men can ask for a psychological visit in case of need. On the other hand Dutch men eligible for AS will *individually meet* with one or several specialists *in sequence*, to talk about RP, RT and AS. Men do not necessarily have to talk to all specialists before deciding on which treatment they would like to start. So while both centers are dedicated AS-centers, the way PCa care is organized in Rotterdam and Milan may add to the different levels of anxiety experienced by AS patients. In the ProtecT trial which randomized PCa patients to either undergoing RP, RT, or active monitoring (an adapted form of AS) lessons have been learned on information provision, decision-making and the role specialists play in that process. It was found that surgeons and oncologists may inadvertently create an additional barrier to AS through their own personal preferences for treatments. On top of that barrier then also comes the difficulty of presenting AS as an equal option to the more traditional definitive treatment options (RP, RT) [18, 25–29]. Such a hesitation may subsequently profile into the patient-physician communication and leave potential marks in the patients’ line of thought.

Our findings, furthermore, revealed that the initial increase of the total MAX-PC and the fear of recurrence scores in Dutch men decreases slowly over time (for the total MAX-PC score with, on average, 1 point for every year on AS) as compared to Italian men which can be explained by the higher overall value that Dutch men reported at the start of AS. The difference in PSA anxiety was however small, and seems to be in the opposite direction of the total MAX-PC and fear of recurrence scores. Eymech et al. studied the biopsychosocial and holistic impact of living with untreated PCa while following an AS strategy through qualitative interviews [30]. It was recognized that men may experience cyclical anxiety around monitoring appointments. So while men after the diagnosis and recognition of the potential impact of low-risk PCa return to a normal state of mind and they do not worry about their PCa on a day-to-day or month-to-month basis, monitoring appointments may still cause a peak-anxiety moment [30].

As mentioned above, country is most likely to represent the way PCa care is organized in the two dedicated AS-centers. It can be hypothesized that once a man enters a clinical organization in which decision making has been collegially implemented, he may feel reassured and “in control” over his health. Moreover, the presence of a psychologist both at the entrance on AS and during the AS monitoring path might be helpful in supporting men and in taking charge of those who feel anxious. Similarly, a previous study from Vasarainen et al. explored the “bridge” role of the nurse between men on AS and the urologist [31]. These findings revealed that receiving support may help in explaining patients the low impact of AS on QoL.

Even though in the Dutch cohort anxiety levels were higher as compared to the Italian cohort, our findings showed that their anxiety levels slowly decreased over time. This is supported by a number of studies which demonstrated that during AS there is a psychological adjustment process ongoing [13, 32], with anxiety decreasing over time [31, 33–35]. Men may feel confident on AS as time goes by, since after being exposed to multiple follow-up AS follow-up visits it may be observed an habituated response effect [36], which in turn may lead to a gradual reduction in anxiety.

There are strengths and limitations in the conduct of this study. A strength is that both the Milan and Rotterdam centre use the PRIAS inclusion criteria and the AS-monitoring scheme. A limitation, however, is the timing of the questionnaires, which were around the same time, but not identical. In the statistical analyses this has been accounted for by including ‘time on AS (in years)’ as a fixed effect. Furthermore, we have to acknowledge that no control group was involved, and that the results of our analyses could hardly be generalized to other countries and/centers. It can, however, be perceived as an invitation to explore this topic further by comparing different countries and different care organizations. Finally, we were not able to distinguish whether the effect of country could also, in part, be attributed to a potential difference in ‘mind set’ between Italian and Dutch men.

## Conclusions

In conclusion, in aiming to identify socio-demographic factors associated with psychological outcome to AS in two dedicated AS-centers being part of the worldwide, multi-center PRIAS study, country seems to be the variable that is associated with the observed differences in levels of anxiety. Country is most likely to represent the way PCa care is organized in the two dedicated AS-centers. In the present study the relevance of a multidisciplinary setting in PCa care organizations is briefly touched upon. A multidisciplinary clinical team including a psychologist can make a difference in offering AS-eligible patients tailored treatment information and in helping them to better understand their choice for AS, which is likely to have an impact on their anxiety levels. In daily clinical practice, clinicians and experts should be aware that involving patients in the decision making phase may easily lead to a higher trust in clinicians, better adherence to treatment recommendations, and facilitate patients’ engagement in quality of healthcare services.

## Data Availability

The datasets used and/or analysed during the current study are available from the corresponding author on reasonable request.

## Abbreviations

PCa: Prostate cancer
RP: Radical prostatectomy
RT: Radiotherapy
AS: Active surveillance
PSA: Prostate-specific antigen
DRE: Digital rectal examination
MRI: Magnetic resonance imaging
QoL: Quality of life
PRIAS: Prostate cancer Research International Active Surveillance study
MAX-PC: Memorial Anxiety scale for Prostate Cancer
IQR: Interquartile range
CI: Confidence interval
EBRT: External beam radiotherapy
BT: Brachytherapy
